# Analytical modeling and FEM simulation of slotted piezoelectric arrays for vibration energy harvesting

**DOI:** 10.1038/s41598-026-69095-0

**Published:** 2026-09-10

**Authors:** Shimaa M. Ahmed, Sallam A. Kouritem, Roaa I. Mubarak, Azza M. Anis

**Affiliations:** 1 Electronics and Communications Engineering Department, Faculty of Engineering, Capital University (Formerly Helwan University), Cairo, Egypt; 2 Interdisciplinary Research Center for Intelligent Manufacturing & Robotics, King Fahd University of Petroleum and Minerals, Dhahran, 31261 Saudi Arabia

**Keywords:** H-Slot Cantilever, Piezoelectric Material, Vibration, Energy Harvester, Finite Element Modeling, Engineering, Materials science, Physics

## Abstract

This work investigates a cantilever-based piezoelectric energy harvester incorporating designs with different slot shapes. The finite element method and analytical modeling are employed to study and compare several slot geometries, including rectangular, triangular, symmetric H, asymmetric H, and inverted triangular designs. The results show that slot geometry and position strongly affect both output power and resonant frequency. The symmetric H-slot delivers nearly double the power output of the solid beam under the same conditions, along with a tenfold increase in voltage. The symmetric H-slot design achieves 0.64 mW at 47 Hz, corresponding to a normalized power density of 0.54 mW·cm⁻³·g⁻²·Hz⁻¹, confirming the effectiveness of the proposed structural modification in enhancing energy harvesting efficiency. Extending the design to slotted cantilever arrays revealed that uniform arrays provide a maximum power output of 1.86 mW, whereas varied-length H-slot arrays sacrifice power for a broader 5.8 Hz bandwidth with stable performance above 50% of peak.

## Introduction

In recent years, the use of compact, low-power devices, especially wireless sensor nodes (WSNs), has expanded rapidly^1–3^. Adoption of medical implants and portable IoT devices has increased significantly in recent years^1,4–7^. To support these applications, power supplies must be reliable and able to operate continuously over long periods without servicing. In many cases, particularly in remote or hard-to-reach locations, replacing or recharging batteries is impractical or even impossible^8–13^. However, the relatively large size of these batteries makes them unsuitable for a wide range of modern applications^14–18^. Recent improvements in MEMS technology and electronic integration have lowered the energy demand of sensing and transmission units to mere hundreds of nano-watts. This challenge has encouraged researchers to focus on self-powered systems that can generate energy from the surrounding environment. Different forms of energy are available, including solar and thermal sources, radio frequency, and mechanical vibrations^19–22^. Among these, vibration-based energy harvesting is very promising because mechanical motion is available in many everyday situations^4,23^. Piezoelectric materials are particularly suitable for this purpose since they can directly convert mechanical strain into electrical energy^24–26^. Energy harvesters that rely on piezoelectric vibrations provide a comparatively higher energy density than many other harvesting techniques^27^. In addition, their operating principle is relatively simple and straightforward to understand^28,29^ and their compact form makes them effective for low-power and MEMS-scale devices. Energy harvesters that operate on vibrations often utilize piezoelectric cantilevers^30^. Cantilevers can produce high strain under base excitation and can resonate at lower frequencies than clamped–clamped beams. Despite these advantages, they still suffer from a main challenge known as the stiffness–frequency–power trade-off. To make the beam resonate at low frequencies (< 50 Hz), which match common vibration sources such as human motion (1–10 Hz) and industrial machines (10–100 Hz)^31–36^. The beam length usually needs to be increased or its stiffness reduced. However, these changes lead to lower stress in the piezoelectric layers, which results in a decrease in the generated power density. Recent studies have shown that optimizing the geometry of the cantilever beam can help overcome the stiffness–frequency–power trade-off and greatly improve the energy harvesting performance^37,38^. Several approaches have been suggested, such as changing the overall shape of the beam, adding internal cavities, or using variable cross-sections to better distribute stress. According to Li et al.^39^, a cantilever-type piezoelectric energy harvester with a curved L-shaped proof mass is proposed. Their results indicated that this configuration lowered the fundamental resonant frequency while improving the power density relative to conventional cantilever designs. Mahreen et al.^40^ emphasized that using a tapered cantilever shape yields a more consistent strain distribution than standard rectangular or trapezoidal beams of equal thickness, thereby increasing the harvested power output. In a related study, Ibrahim et al.^41^ conducted a comparison between beams tapered in width and those tapered in thickness, concluding that thickness tapering achieves better output power than width tapering.

Baker et al.^42^ examined several cantilever geometries to enhance power generation. Their results showed that trapezoidal beams produced up to 30% more power per unit volume than standard rectangular ones. Xu et al.^43^ designed a trapezoidal hollow cantilever that lowered the resonant frequency by 12.18% and increased the output voltage by 34.67%. Săvescu et al.^44^ proposed a triangular cantilever beam that produced nearly the same power as the rectangular type but used 35% less piezoelectric material, leading to higher power density. Yang et al.^45^ proposed an arc-shaped harvester, reporting that it produced between 2.55 and 4.25 times greater power than standard flat designs. Furthermore, its geometry resulted in a lower resonant frequency, which makes it advantageous for capturing energy from low-frequency vibrations. Karadag et al.^46^ optimized the cantilever geometry by adopting a curved profile. Their findings revealed that, with or without a 5 g tip mass, the new design outperformed the triangular beam, improving strain uniformity by approximately 22% and 29%, respectively. Wang et al.^47^ used trapezoidal cavities to reach a power density of 8.93 mW/cm³. Similarly, Mohiuddin et al.^48^ worked on partially coated and inverted trapezoidal beams to maximize power density. Other researchers have studied the effect of changing the tip mass geometry. Chen and Bedekar^49^ showed that altering the cantilever structure, especially with a two-part trapezoidal layout, considerably boosted the harvested power.

Previous studies have shown that the shape of slots can significantly influence the stress distribution in beam structures^50^. However, this study was not conducted on piezoelectric energy harvesters. In the field of piezoelectric energy harvesting, Xiong et al.^51^ and Tamrakar et al.^52^ introduced a rectangular slot to improve the performance of piezoelectric cantilevers. Although these studies reported improved strain distribution and reduced resonant frequency, they were limited to a single slot geometry and did not analyze the effect of slot shape on the other design metrics.

In this study, a finite element analysis (FEA) is conducted to investigate the performance of a piezoelectric cantilever beam with different slot shapes. Various slot configurations, including rectangular, symmetric H, asymmetric H, triangular, and inverted triangular slots, are introduced into the cantilever. In addition, the effects of slot position and dimensions are analyzed through the FEA to maximize output power. Furthermore, the impact of slot shape on stress distribution is evaluated at the resonant condition. A comparative study is performed on the beams with different slot shapes. The results demonstrated that cantilevers with symmetric H-slots enhance and broaden the stress distribution across the piezoelectric layer, leading to improved energy harvesting performance. Furthermore, the proposed slot shapes are evaluated not only in single harvesters but also in array configurations to investigate their potential for both power enhancement and bandwidth extension.

## Modeling

**Fig. 1:**
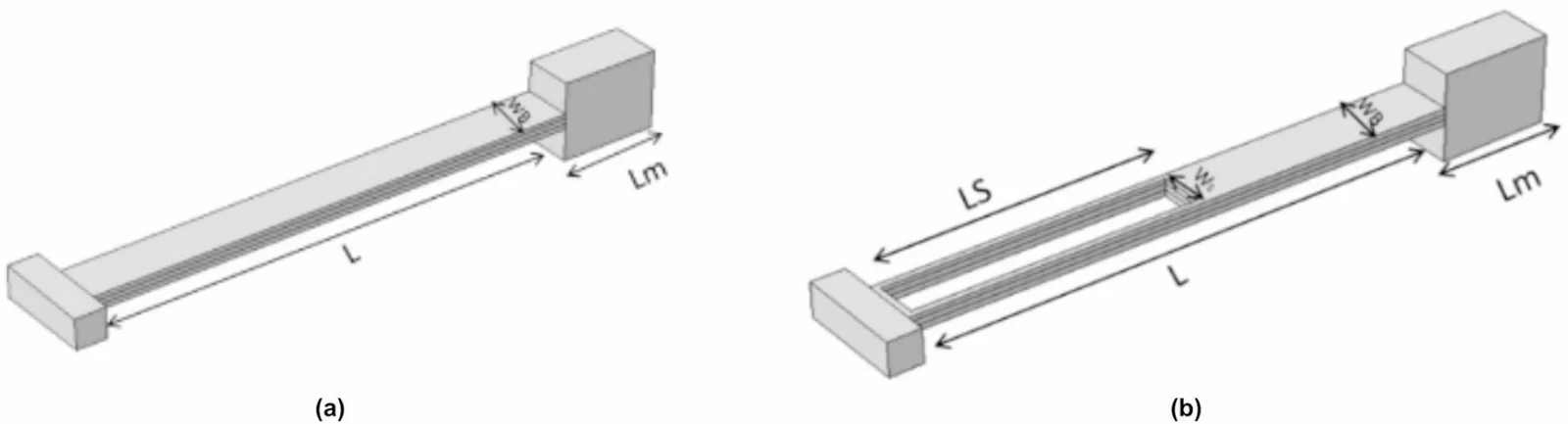
Structure of (**a**) standard cantilever and (**b**) cantilever with rectangular slot.

To investigate the system response, the analytical and FEA models are developed. The analytical formulation provides closed-form expressions for the dynamic behavior and electrical output under harmonic excitation, allowing for direct insight into the governing parameters. In parallel, the FEA model is employed to capture more complex effects, including geometric modifications, stress distribution, and localized strain concentrations within the piezoelectric layers. Together, these two approaches provide a comprehensive framework that enables the validation of theoretical predictions against numerical simulations and ensures the reliability of the performance assessment. Figure 1 illustrates the two configurations of the proposed piezoelectric cantilever energy harvester. In both configurations, the harvester consists of a cantilever beam with a proof mass attached to its free end and a fixed support at the opposite end. A metallic substrate is bonded between two piezoelectric layers, forming a bimorph structure. The two piezoelectric layers are electrically connected in parallel. A bimorph configuration is used because it provides stronger electromechanical coupling and higher energy conversion than a single piezoelectric layer under the same mechanical excitation^53^. The two piezoelectric layers are connected in parallel to increase the equivalent capacitance and the available output current while maintaining approximately the same output voltage as a single layer. Although a series connection produces a higher output voltage, it also requires a substantially higher optimal load resistance, whereas the maximum harvested power under impedance-matched conditions remains nearly unchanged^54^. Therefore, the parallel configuration is selected as the more practical choice for the present study. A proof mass is attached to the free end of the beam. The structure is excited by an acceleration source applied at the clamped end. Figure 1(a) illustrates the conventional solid cantilever, whereas Fig. 1(b) shows the modified cantilever incorporating a rectangular slot.

**Fig. 2:**
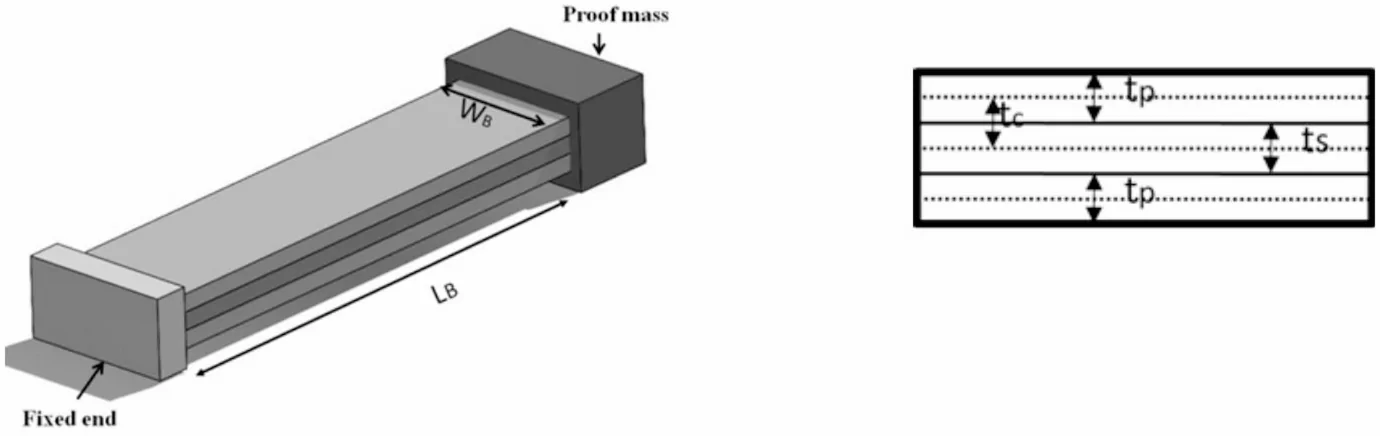
Cross-section parameters of the standard cantilever.

### Analytical model

Device performance is governed by its structural parameters, with primary metrics including eigenfrequency, output voltage, and harvested power. To improve the output performance, the structural parameters that affect it are adjusted, and the relationship between these parameters and the performance is studied. For analysis, the geometry from Fig. 1 is reduced to the simplified configuration illustrated in Fig. 2. In this figure, the x-axis is defined along the beam’s length. The displacement of the tip mass at the free end is denoted by z(t) when the base is subjected to an external acceleration y¨(t). Key geometric parameters include the beam length $\:\left(L\right)$, the horizontal span to the tip mass$\:{\:L}_{B}$, and the tip mass dimensions $\:{\:L}_{m}$, $\:{\:t}_{m}$, with mass m_t_.

**Fig. 3:**
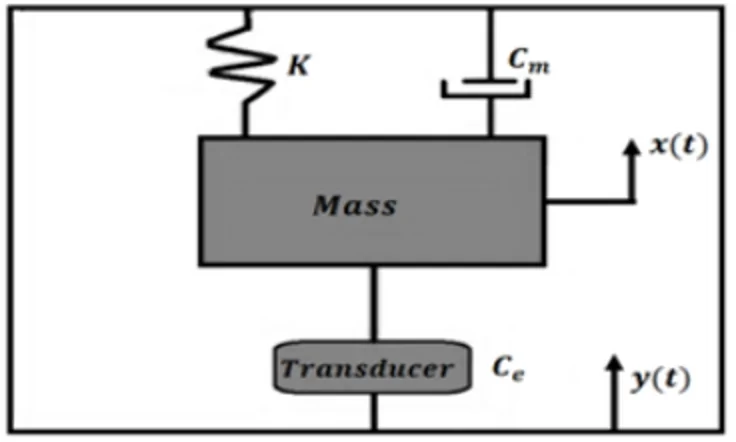
Spring-based mass-damping model.

The central metallic substrate (length $\:{\:L}_{B}$, thickness $\:{\:t}_{s}$) includes a rectangular slot of length $\:{\:L}_{slot}$. The upper and lower piezoelectric layers (length $\:{\:L}_{B}$, thickness $\:{h}_{c}$) share a uniform width $\:{\:w}_{B}$, with the slot width $\:{\:w}_{slot}$locally reducing the cross-section. The harvester dynamics are modeled using a single-degree-of-freedom (SDOF) mass–spring–damper system subjected to base excitation, as shown in Fig. 3.

It consists of a mass M supported by a spring with stiffness K, a damping coefficient C, and a resonant frequency ω_n_. When the generator is subjected to vibration with y(t), the mass oscillates out of phase with the generator housing with z(t), creating a relative motion between them. This displacement has a sinusoidal amplitude and can be used to drive a suitable transducer for generating electrical energy. Within this framework, the tip mass obeys the standard second-order motion equation with electromechanical coupling, where inertia, damping, stiffness, coupling, and base acceleration appear explicitly^55^:

1 $$\:m\:\ddot{z\left(t\right)}+\:{b}_{e}\dot{z\left(t\right)}+k\:z\left(t\right)+\theta\:v\left(t\right)=m\ddot{y\left(t\right)}$$

2 $$\:\ddot{z\left(t\right)}+\:2{\xi\:}_{m}{\omega\:}_{n}\dot{z\left(t\right)}+{{\omega\:}_{n}}^{2}\:z\left(t\right)+\frac{\theta\:}{m}v=\ddot{y\left(t\right)}\:$$

where $\mathop {mz(t)}\limits^{{ \cdot \cdot }}$ represents the inertial force of the mass as it moves relative to the vibrating base, b_e_ z˙(t) is the mechanical damping force, proportional to velocity, which dissipates energy and reduces motion, k z(t) represents the restoring force generated by the spring or cantilever, acting to return the mass to its equilibrium position, θ$\:\:v$ (t) represents the electromechanical coupling force from the piezoelectric element, which depends on the output voltage, and my¨(t) represents the excitation force resulting from the base vibration, which drives the system. The equivalent electrical circuit, which incorporates the electromechanical coupling, can be derived based on the piezoelectric constitutive relations^56^:

3 $$\:\left\{S\right\}=\left[{s}^{E}\right]\left\{T\right\}+{\left[e\right]}^{t}\left\{E\right\}$$

4 $$\:\left\{D\right\}=\left[e\right]\left\{S\right\}+\left[{\epsilon\:}^{T}\right]\left\{E\right\}$$

where {S} represents the six-component strain vector, while {T} denotes the corresponding stress vector. The electric displacement is given by the three-component vector {D}, and {E} stands for the electric field vector. The matrix [s^E^] is a 6 × 6 compliance matrix evaluated under constant electric field conditions. The piezoelectric stress coefficient is described by the [e] matrix, which is 3 × 6 and contains the piezoelectric strain coefficients. [ε^t^] is a 3 × 3 dielectric permittivity matrix defined at constant mechanical stress. A two-layer bending element configured as a cantilever beam, as illustrated in Fig. 1, is considered in this analysis. Consistent with typical bending configurations, the piezoelectric material is poled along the z-axis, and electrodes are positioned on the surfaces perpendicular to this axis. The excitation is assumed to occur solely in the 3-direction. Under these conditions, the piezoelectric layer primarily experiences a one-dimensional stress state along the 1-axis. As a result, the general piezoelectric constitutive equations simplify to their reduced form, as shown below:

5 $$\:{T}_{1}={{{c}^{E}}_{11}S}_{1}-{e}_{31}{E}_{3}$$

6 $$\:{D}_{3}={e}_{31}{S}_{1}+{{\epsilon\:}^{s}}_{33}{E}_{3}$$

where T_1_ denotes the mechanical stress, S1 is the corresponding strain, c_11_^E^ represents Young’s modulus under a constant electric field, and e_31_ is the piezoelectric stress coefficient. E_3_ stands for the electric field, D_3_ is the electric displacement, and ε_33_^S^ refers to the material’s permittivity under constant strain. Based on these definitions, Eq. (6) can be rewritten as:

7 $$\:{D}_{3}={d}_{31}{T}_{1}+{{\epsilon\:}^{s}}_{33}{E}_{3}$$

The stress distribution $\:T\left(x\right)$ within the piezoelectric layer along the length of the beam can be expressed as^57^:

8 $$\:T\left(x\right)=\left\{\frac{M\left(x\right)}{I}\right\}{t}_{ps}$$

* I* is the effective moment of inertia of the standard bimorph cantilever, which can be expressed in Eq. (9) below:

9 $$\:{I}_{eff}={I}_{Beam}=2\left[\frac{{{t}_{p}}^{3}{w}_{B}}{12}+{w}_{B}{{t}_{c}}^{2}{t}_{p}\right]+\frac{\tau\:\:{w}_{B}{{t}_{s}}^{3}}{12}$$

The analytical model is intentionally derived under the assumptions of an equivalent Euler–Bernoulli beam to obtain a tractable closed-form electromechanical model. In the proposed structure, inserting a slot in the beam will modify the stiffness of the beam^58^:

10 $$\:{I}_{eff}={I}_{Beam}-{I}_{slot}$$

Where $\:{I}_{slot}$ represents the moment of inertia corresponding to the slot and can be formulated as:

11 $$\:{I}_{slot}=2\left[\frac{{{t}_{p}}^{3}{w}_{slot}}{12}+{w}_{slot}{t}_{p}{{t}_{c}}^{2}\right]+\frac{\tau\:\:{w}_{slot}{{t}_{s}}^{3}}{12}$$

It should be noted that Eq. (10) provides an equivalent representation of the global bending stiffness of the slotted cantilever. Consequently, local stress concentrations and geometry-dependent stress variations are not fully reflected in this simplified analytical formulation. These effects are evaluated using the finite element model, which incorporates the actual slot geometry.

In this context, $\:{W}_{B}$ represents the beam’s width, while * τ * is defined as the ratio of the substrate’s elastic constant to that of the piezoelectric layer ($\:{\uptau\:}$ =c_s_/c_p_). Here, $\:{\:c}_{p}\:$ refers to the elastic constant of the piezoelectric material, and$\:{\:c}_{s}\:$corresponds to that of the substrate. The other parameters are illustrated in Fig. 2. $\:M\left(x\right)$ denotes the bending moment generated by the motion of the end mass, which can be expressed as^59^:

12 $$\:M\left(x\right)={F}_{in}({L}_{B}+\left(\frac{{L}_{m}}{2}\right)-x)$$

where$\:{\:{F}_{in}}_{\:}$represents the force generated by the motion of the end mass, which is defined as the product of the spring constant * k* and the relative displacement $\:z\:$of the mass^60^.

Based on Eqs. (9) and (12), Eq. (8) can be rewritten as:

13 $$\:T\left(x\right)=\frac{k\:z\:({L}_{B}+\left(\frac{{L}_{m}}{2}\right)-x)}{{I}_{eff}}{t}_{c}$$

Equation (13) provides a simplified analytical expression for the longitudinal bending stress by representing the slotted cantilever as an equivalent beam with a constant effective moment of inertia, $\:{I}_{eff}$. The beam’s overall stress average, denoted by * T*, is evaluated using the integration of Eq. (13) along the beam^59^,

14 $$\:{T}_{1}=\frac{1}{{L}_{E}}{\int\:}_{0}^{{L}_{E}}\frac{M\left(x\right){t}_{c}}{{I}_{eff}}\:dx$$

15 $$\:{T}_{1}=\frac{kz({L}_{B}+{L}_{m})}{2{I}_{eff}}{t}_{c}$$

le represents the electrode length that overlays the piezoelectric layer. Although the electrode does not necessarily extend across the entire beam, in this model, it is assumed that $\:{l}_{e}\:=\:{l}_{B}$, meaning it extends over the full beam length. Substituting Eq. (15) into Eq. (7) gives:

16 $$\:{D}_{3}={d}_{31}\frac{kz({L}_{B}+{L}_{m})}{2{I}_{eff}}{t}_{c}+{{\epsilon\:}^{s}}_{33}{E}_{3}$$

The charge produced on the electrode is generated through the direct piezoelectric effect

17 $$\:q={D}_{3}{W}_{B}{L}_{B}={d}_{31}\left[\frac{kz\left({L}_{B}+{L}_{m}\right)}{2{I}_{eff}}{t}_{c}+{{\epsilon\:}^{s}}_{33}{E}_{3}\right]{W}_{B}{L}_{B}-{{\epsilon\:}^{s}}_{33}{E}_{3}{W}_{B}{L}_{B}$$

The voltage difference across the two-layer bender is described with respect to the electric field as^58,61^:

18 $$\:E=\frac{\:v}{{t}_{p}}$$

Then Eq. (17) can be rewritten as:

19 $$\:q={D}_{3}{W}_{B}{L}_{B}={d}_{31}\left[\frac{kz\left({L}_{B}+{L}_{m}\right)}{2I}{t}_{c}+{{\epsilon\:}^{s}}_{33}{E}_{3}\right]{W}_{B}{L}_{B}-{{\epsilon\:}^{s}}_{33}\frac{v}{{t}_{p}}{W}_{B}{L}_{B}$$

The load resistance current is determined using the relation below:

20 $$\:{i}_{Rl}=\frac{dq}{dt}=\frac{v}{Rl}={d}_{31}\left[\frac{k\:{t}_{c}\left({L}_{B}+{L}_{m}\right)}{2I}\frac{dz}{dt}\right]{W}_{B}{L}_{B}-{{\epsilon\:}^{s}}_{33}\frac{{W}_{B}{L}_{B}}{{t}_{p}}\frac{dv}{dt}$$

By rearranging the terms, Eq. (20) can be written as:

21 $$\:\frac{v}{Rl}+{{\epsilon\:}^{s}}_{33}\frac{{W}_{B}{L}_{B}}{{t}_{p}}\frac{dv}{dt}{=d}_{31}\left[\frac{k\:{t}_{c}{W}_{B}{L}_{B}\left({L}_{B}+{L}_{m}\right)}{2I}\right]\frac{dz}{dt}$$

Comparing Eq. (21) with the electrical performance of the piezoelectric harvesting system^62^, the capacitance of the piezoelectric ($\:{c}_{p})$and the induced damping coefficient (ξ_e_ ):

22 $$\:{{\upxi\:}}_{\mathrm{e}}{=d}_{31}\left[\frac{k\:{t}_{c}{W}_{B}{L}_{B}\left({L}_{B}+{L}_{m}\right)}{2I}\right]$$

23 $$\:{c}_{p}={{\epsilon\:}^{s}}_{33}\frac{{W}_{B}{L}_{B}}{{t}_{p}}\:\:\:\:\:\:\:\:\:\:\:\:\:\:\:\:\:\:\:\:\:\:\:\:\:\:\:\:\:\:\:\:\:\:\:\:\:\:\:\:\:\:\:\:\:\:\:\:\:\:\:\:\:\:\:\:\:\:$$

In this case, the piezoelectric harvester contains two layers of piezoelectric material, which are connected in parallel; the equivalent capacitance doubles, and also the electrically induced damping coefficient is doubled. Now we can write the electromechanically coupled governing equations of the system:

24 $$\:\frac{v\left(t\right)}{{R}_{l}}+2{c}_{p}\frac{dv\left(t\right)}{dt}-2{{\upxi\:}}_{\mathrm{e}}\frac{\mathrm{d}\mathrm{z}\left(\mathrm{t}\right)}{\mathrm{d}\mathrm{t}}=0\:\:\:\:\:\:\:\:\:\:\:\:\:\:\:\:\:\:\:\:\:\:\:\:\:\:\:\:\:\:\:\:\:\:\:\:\:\:\:\:\:\:\:\:\:\:$$

25 $$\:\ddot{z\left(t\right)}+\:2{\xi\:}_{m}{\omega\:}_{n}\dot{z\left(t\right)}+{{\omega\:}_{n}}^{2}\:z\left(t\right)+\frac{2\theta\:}{m}v=\ddot{y\left(t\right)}$$

In the case of a rectangular cantilever energy harvester equipped with a rectangular proof mass subjected to vertical harmonic excitation, the resonant frequency is determined from the equivalent stiffness of the system and can be expressed as^63^:

26 $$\:{K}_{eq}=\frac{3Y{I}_{eff}}{{{L}_{B}}^{3}}\:\:$$

The overall stiffness of the composite beam is determined by the combined contributions of the substrate beam and the bonded piezoelectric layer^64^:

27 $$\:YI={Y}_{Sub}{I}_{sub}+2{Y}_{piezo}{I}_{piezo}$$

The resonant frequency of a rectangular beam and a tip mass can be calculated by^65^:

28 $$\:{\omega\:}_{n}=\sqrt{\frac{{K}_{eq}}{{m}_{eff}}}$$

where ω_n_ = 2πf_n_ is the angular resonant frequency in (rad/s), and f_n_ is the resonant frequency (Hz), and m_eff_ corresponds to the effective mass of the combined beam and tip mass, which can be calculated by:

29 $$\:{m}_{eff}=0.24{m}_{b}+{m}_{t}$$

The resonant frequency of the piezoelectric energy harvester is expressed as:

30 $$\:{\omega\:}_{n}=\sqrt{\frac{3Y{I}_{eff}}{{m}_{t}+0.24{m}_{b}}}$$

Solving Eqs. (24) and (25) yields the system’s dynamic response. The end mass motion and the output voltage generated are considered harmonic in nature:

31 $$\:z\left(t\right)=Z{e}^{j\omega\:t}$$

32 $$\:v\left(t\right)=V{e}^{j\omega\:t}\:\:\:\:\:\:\:\:\:\:\:\:\:\:\:\:\:\:\:\:\:\:\:\:\:\:\:\:\:\:\:\:\:\:\:\:\:\:\:\:\:\:\:\:\:\:\:\:\:\:\:\:\:\:\:\:\:\:\:\:\:\:\:$$

The frequency domain relations can be written:

33 $$\:V+j\omega\:2{R}_{l}{c}_{p}V-j\omega\:2{R}_{l}{\xi\:}_{e}\:Z=0\:\:\:\:\:\:\:\:\:\:$$

34 $$\:-{\omega\:}^{2}Z+2j\omega\:{\omega\:}_{n}{\xi\:}_{m}\:Z+{{\omega\:}_{n}}^{2}Z+2\frac{{\xi\:}_{e}}{m}V=-{\omega\:}^{2}Y$$

Substituting Eq. (33) into Eq. (34) gives:

35 $$\:Z=\frac{-{\omega\:}^{2}Y\left(1+j\omega\:2{R}_{l}{c}_{p}\right)}{(-{\omega\:}^{2}+2j\omega\:{\omega\:}_{n}{\xi\:}_{m}\:+{{\omega\:}_{n}}^{2})\left(1+j\omega\:2{R}_{l}{c}_{p}\right)+4\frac{j\omega\:{R}_{l}{{\xi\:}_{e}}^{2}}{m}}$$

36 $$\:V=\frac{-j{\omega\:}^{3}2{R}_{l}{\xi\:}_{e}Y}{(-{\omega\:}^{2}+2j\omega\:{\omega\:}_{n}{\xi\:}_{m}\:+{{\omega\:}_{n}}^{2})\left(1+j\omega\:2{R}_{l}{c}_{p}\right)+4\frac{j\omega\:{R}_{l}{{\xi\:}_{e}}^{2}}{m}}$$

After determining $\:v\left(t\right)$, the power delivered to the load can be obtained using

37 $$\:P=\frac{{V}^{2}}{{R}_{l}}$$

### FEA for proposed cantilever

**Fig. 4:**
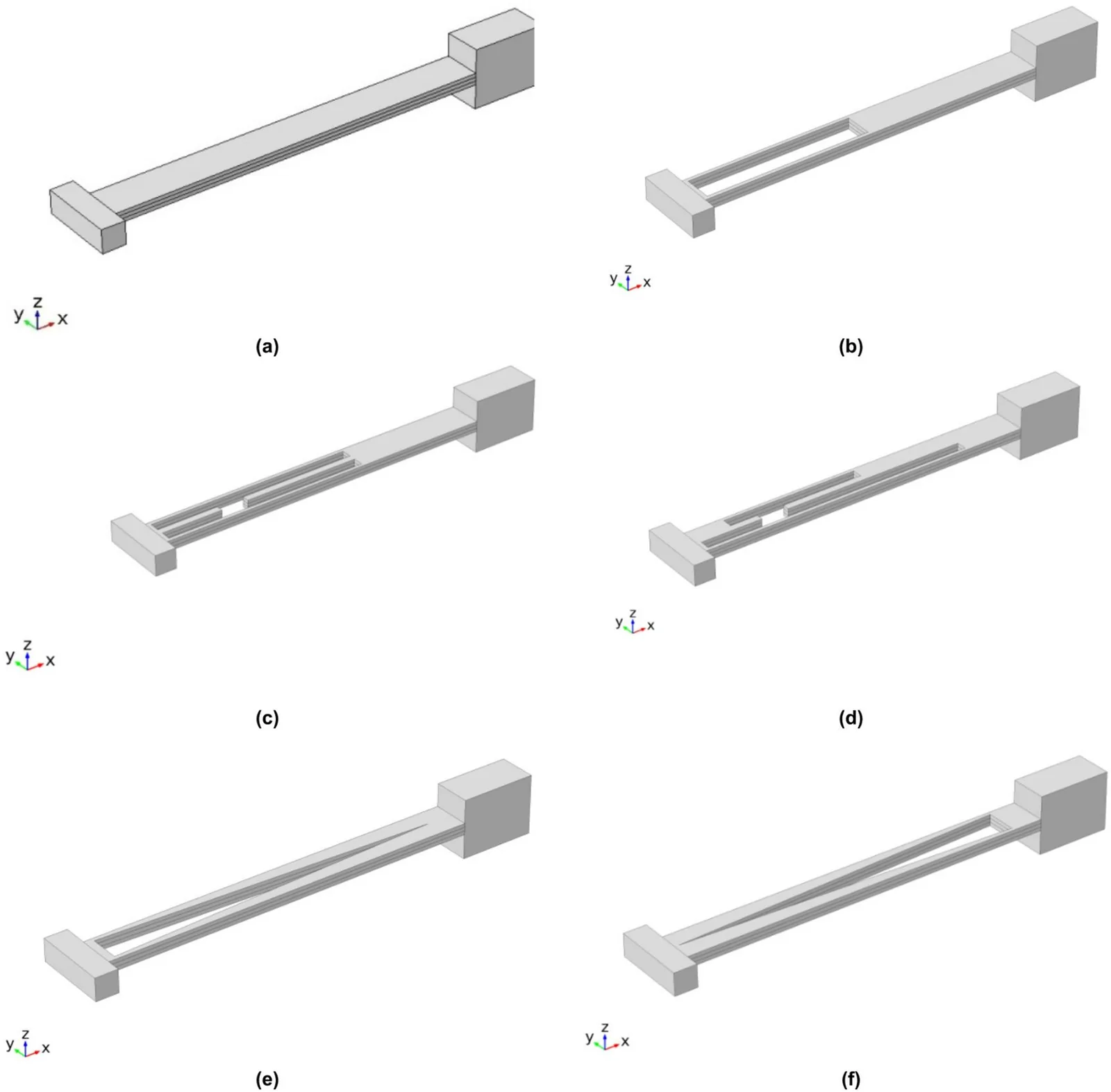
Structure of the cantilever with (**a**) no slots, (**b**) a rectangular slot, (**c**) a symmetric H-slot, (**d**) an asymmetric H-slot, (**e**) a triangle slot, and (**f**) an inverted triangle slot.

This work develops different slot geometries, as shown in Fig. 4. In all designs, the external beam dimensions, the piezoelectric layers, the proof mass, the material properties, the removed material volume, and the boundary conditions are identical, while the slot shape is varied. The analysis is primarily conducted numerically. The simulations are carried out in COMSOL Multiphysics using a combination of solid mechanics, electrostatics, and electrical circuit modules. The solid mechanics module is responsible for capturing the structural response of the cantilever, including vibration-induced deformations and stresses. In the solid mechanics module, body loads are applied to represent external vibrations. The acceleration-based body load is derived starting from the fundamental relation between force, mass, and acceleration. For each element, the applied force$\:\:{F}_{\mathrm{a}\mathrm{p}\mathrm{p}\mathrm{l}\mathrm{i}\mathrm{e}\mathrm{d}}$ is related to its mass m and acceleration a, as

38 $$\:{F}_{applied}=m.a$$

Concurrently,

39 $$\:m=\rho\:\:v$$

where $\:\rho\:$ denotes the density, while * v* is the volume of the corresponding element. The applied acceleration is defined as

40 $$\:a=acc\:.g.\mathrm{sin}\left(2\pi\:ft\right)$$

where acc is the acceleration coefficient,$\:\:g$ is the gravitational acceleration (1 g = 9.795 m/s^2^), f is the excitation frequency, and t is the vibration time. Accordingly, the force acting on each element is obtained as

41 $$\:{F}_{applied}=m.acc\:.\:\rho\:\:.v\:.g\mathrm{sin}\left(2\pi\:ft\right)$$

Thus, the force applied to the model per unit volume can be written as:

42 $$\:{F}_{applied}=m.acc\:.\:\:\rho\:.g\mathrm{sin}\left(2\pi\:ft\right)$$

An excitation equivalent to 0.2 g is imposed on the base structure, including the substrate, piezoelectric layers, and the attached tip mass. The electrostatics module is employed to simulate the electric field distribution within the piezoelectric layer, enabling the estimation of the potential generated due to mechanical strain. The electric circuit module is then integrated to represent the connection with an external load, allowing the evaluation of power output under vibrational excitation. Tetrahedral elements are chosen for meshing as they provide the most appropriate discretization for the model. An eigenfrequency study is first carried out to determine the fundamental resonant frequency (*n* = 1), as this mode is directly associated with maximum energy output. Following this, a frequency-domain study is performed to assess the variation of power output around the first eigenmode. A damping ratio of 5% is considered in all structures. The output power is formulated in FEM as a function of electrical resistance, base excitation, resonant frequency, inertial mass, damping coefficient, and capacitance. To identify the optimal slot shape for maximizing the harvested power, different slot configurations are systematically investigated while keeping the removed material volume constant.

A simple rectangular bimorph cantilever is analyzed with dimensions: beam length L_B_, width W_B_, and thickness of both piezoelectric and substrate layers $\:{t}_{p}={t}_{s}$. A tip mass of 3 g is attached to the free end. The device is fabricated using brass for the substrate and PZT-5 H for the piezoelectric layer. The piezoelectric coefficient matrix, elastic stiffness matrix, and dielectric constant matrix for PZT-5 H are presented in Eqs. (43)–(45), respectively.

43 $$\:d=\left\{\begin{array}{cc}\begin{array}{ccc}0&\:0&\:0\\\:0&\:0&\:0\\\:-1.86&\:-1.86&\:6.7\end{array}&\:\begin{array}{ccc}0&\:6.6&\:0\\\:6.6&\:0&\:0\\\:0&\:0&\:0\end{array}\end{array}\right\}\:\times\:{10}^{-10}$$

45 $$\:{\epsilon\:}_{r}=\left\{\begin{array}{ccc}3130&\:0&\:0\\\:0&\:3130&\:0\\\:0&\:0&\:3400\end{array}\right\}$$

The material properties of both PZT-5 H and brass are listed in Table 1. The performance metric adopted in this study is the output power, expressed as the generated power in mW. The power harvesting equation employed in the COMSOL simulations for all models in this study follows the formulation of Lefeuvre et al.^66^. They proposed a comprehensive model applicable to both linear and nonlinear harvesters with variable width. The generalized expression for the harvested power in terms of these parameters is given as:

46 $$\:Power=\frac{\:R\:{{\alpha\:}_{2}}^{2}{{{\omega\:}_{n}}^{4\:}{U}^{2}M}^{2}}{{{\left(\frac{\pi\:}{2}+R{c}_{p}{\omega\:}_{n}\right)}^{2}\left({C}_{v}+\left(2R\:{{\alpha\:}_{2}}^{2}/\left(R{c}_{p}{\omega\:}_{n}+{\left(\frac{\pi\:}{2}\right)}^{2}\right)\right)\right)}^{2}}\:$$

In its reduced form, the output power is represented as:

47 $$\:Power={V}_{re\:}\left(\frac{2{\alpha\:}_{2}}{\frac{\pi\:}{2}+{R}_{l}{c}_{p}{\omega\:}_{n}}+{C}_{v}\frac{\frac{\pi\:}{2}+{R}_{l}{c}_{p}{\omega\:}_{n}}{{\alpha\:}_{2}{R}_{l}}\right)\:$$

here α_2_ denotes the electromechanical coupling coefficient, c_p_ is the capacitance (C/V), $\:{V}_{re}$ represents the voltage (V), and $\:{c}_{v}$ is the damping coefficient arising from strain and boundary effects (N.s/m). M refers to the dynamic mass (kg), * U* is the base excitation displacement (m), Uωn^2^ corresponds to the base acceleration (m/s²), ω_n_ is the angular resonant frequency (rad/s), and $\:{R}_{l}$ is the load resistance (Ω).

**Table 1:** Dimensions and material properties used in the simulation.

Description	Value
Beam length (L)	50 mm
Beam width (W)	5 mm
Substrate thickness (h_s_)	0.5 mm
Piezoelectric thickness (h_p_)	0.5 mm
Tip mass length (L_T_)	10 mm
Tip mass width (W_T_)	5 mm
Tip mass thickness (h_T_)	7.8 mm
Young’s modulus of substrate (Y_s_)	110 GPa
Young’s modulus of piezoelectric (Y_p_)	60.6 GPa
Density of substrate material (brass)	9000 kg/m^3^
Density of tip mass material (brass)	9000 kg/m^3^
Density of piezoelectric material (PZT-5 H)	7500 kg/m^3^

### Results of proposed cantilever

**Fig. 5:**
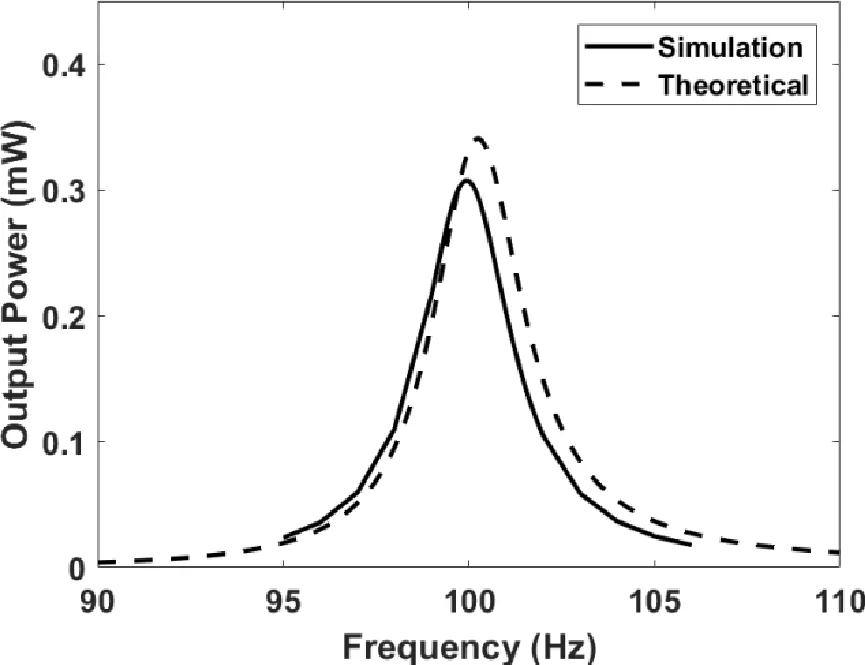
Comparison between analytical results and COMSOL simulations with the reference cantilever reported by Kundu and Nemade^67^.

To verify the accuracy of the proposed analytical model and to ensure consistency with earlier works, the reference cantilever geometry reported by Kundu and Nemade^67^ is first selected as a benchmark case. In their study, a simple rectangular bimorph cantilever is analyzed with the following dimensions: beam length$\:{L}_{B}=50$ mm, width $\:{W}_{B}=5$ mm, and thickness of both piezoelectric and substrate layers $\:{t}_{p}={t}_{s}$= 0.5 mm. A tip mass of 10 × 5 × 7.8 mm³ is attached with 3 g, and the device is fabricated using PZT-5 H for the piezoelectric layer and brass for the substrate. In the present study, the same geometry is modeled and simulated in COMSOL Multiphysics to establish a validation baseline. The numerical model is examined for output power by sweeping the excitation frequency in the range of 90–110 Hz under a base acceleration of 0.2 g. The maximum output power of 0.32 mW is obtained at 100 Hz, which corresponds to the resonance frequency of the system, as illustrated in Fig. 5. The validation results show very close agreement with the published data, with a fundamental resonant frequency at 100 Hz and a power difference of less than 3%, demonstrating the reliability of the developed framework. A direct comparison between the output power calculated from the analytical model of this work^67^ and that obtained from COMSOL is presented in Fig. 5, where the two curves overlap closely.

**Fig. 6:**
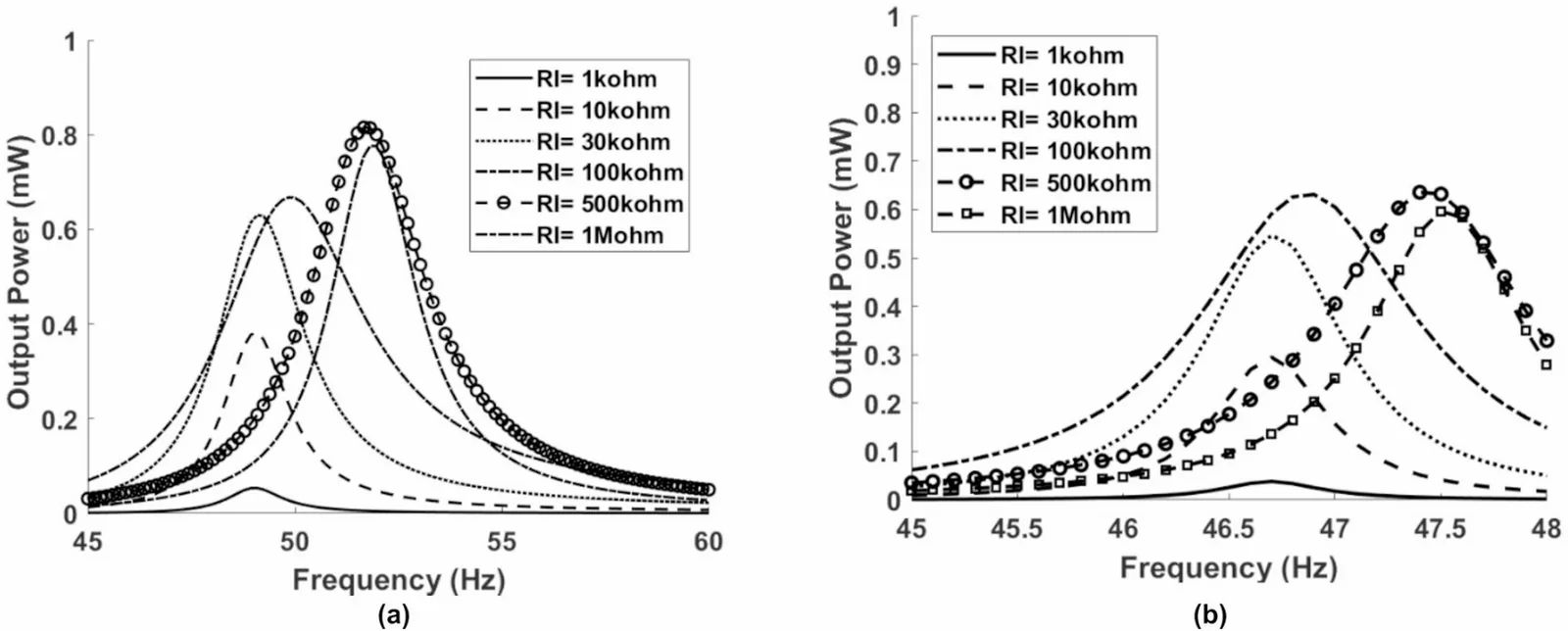
Output power versus frequency for the cantilever with a rectangular slot: (**a**) the proposed analytical model and (**b**) COMSOL simulation.

Building on this validated reference case, the work is extended to include a rectangular slot geometry introduced into the cantilever. The analytical results presented in Sect.  3.1 for a cantilever with a rectangular slot are compared with finite element simulations performed in COMSOL Multiphysics for the same structure. The mathematical model is implemented in MATLAB to determine the harvested power at different load resistances, which is then calculated and plotted against the excitation frequency, as shown in Fig. 6.

The harvested power of the proposed rectangular-slotted cantilever depends on the external load resistance and reaches a maximum at an optimum resistance value. In the finite element analysis, the optimum load resistance is determined from the load resistance sweep shown in Fig. 6(b) by identifying the resistance corresponding to the maximum output power. In the analytical model, the optimum load resistance is obtained by differentiating the resonant power expression with respect to R_L_ and setting the derivative equal to zero. Both approaches yielded the same optimum load resistance of 500 kΩ.

**Fig. 7:**
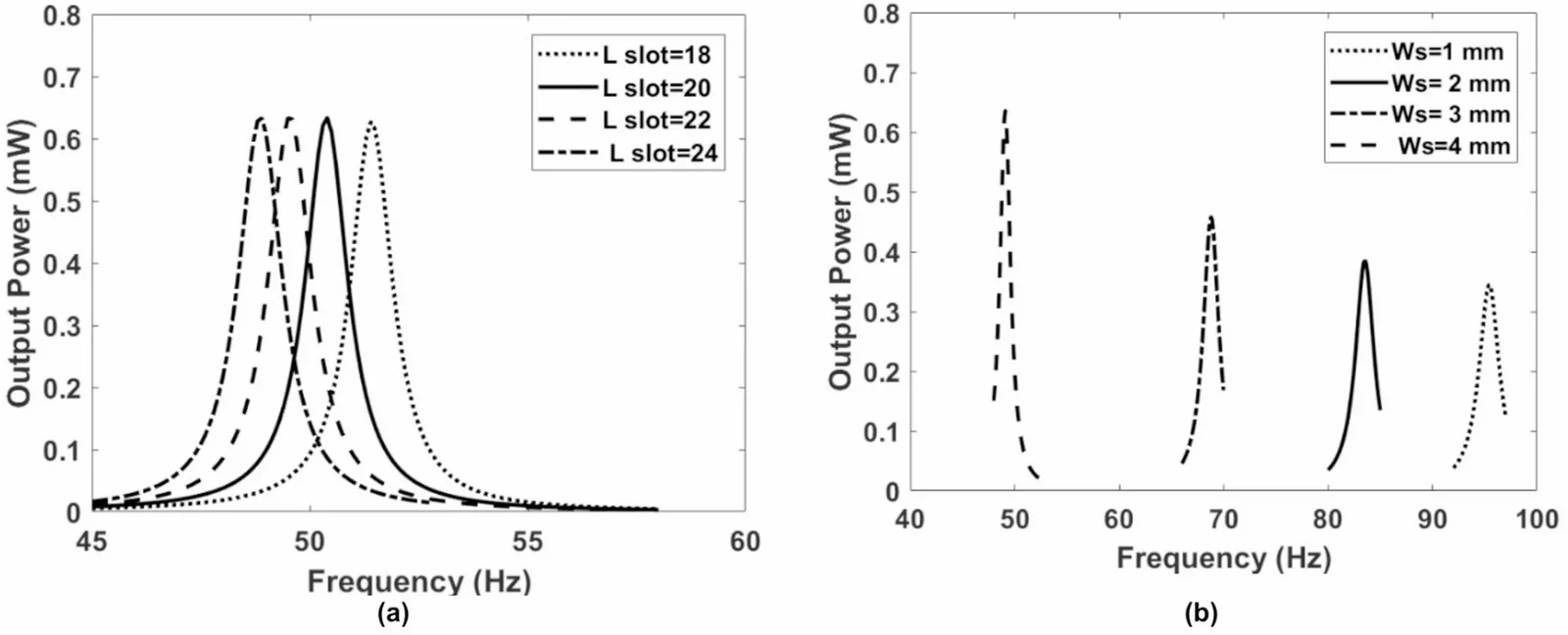
Effect of slot dimensions on the resonant frequency and output power of the cantilever beam: variation with (**a**) slot length, and (**b**) slot width.

At this operating condition, the analytical model predicts a harvested power of 0.8 mW, whereas the finite element simulation predicts 0.64 mW. The agreement in the optimum load resistance supports the analytical prediction, while the difference in the harvested power is mainly attributed to the simplifying assumptions adopted in the analytical model. In contrast, the finite element model accounts for the actual slot geometry and the coupled electromechanical response of the cantilever, resulting in a more accurate prediction of the harvested power. After that, building on this validated reference case, the work is extended to include different slot geometries introduced into the cantilever substrate. The slot designs considered in this study are rectangular, triangular, symmetric H-slot, and asymmetric H-slot. The purpose of these modifications is to alter the neutral axis and redistribute the stress field along the beam, which increases the strain experienced by the piezoelectric layer. This mechanism enhances the electromechanical conversion efficiency without increasing the device volume. The effects of slot length and width on the output power and resonant frequency are investigated through individual parametric sweeps performed in COMSOL Multiphysics. In this investigation, the rectangular slot length is varied between 18 mm and 24 mm, as illustrated in Fig. 7(a). The results show that increasing the slot length leads to a reduction in the resonant frequency of the beam, while the output power remains nearly constant. Also, the rectangular slot width is varied between 1 mm and 4 mm, as illustrated in Fig. 7(b). The results show that increasing the slot width leads to a reduction in the resonant frequency of the beam, while the output power is increased. The results obtained from Fig. 7 show that the slot width is the more effective parameter than slot length due to the effective moment of inertia depends on slot width according to Eqs. (10) and (11). This indicates that widening the slot decreases the effective moment of inertia, which lowers the resonant frequency. At the same time, electrical damping increases because it is inversely related to the moment of inertia.

**Fig. 8:**
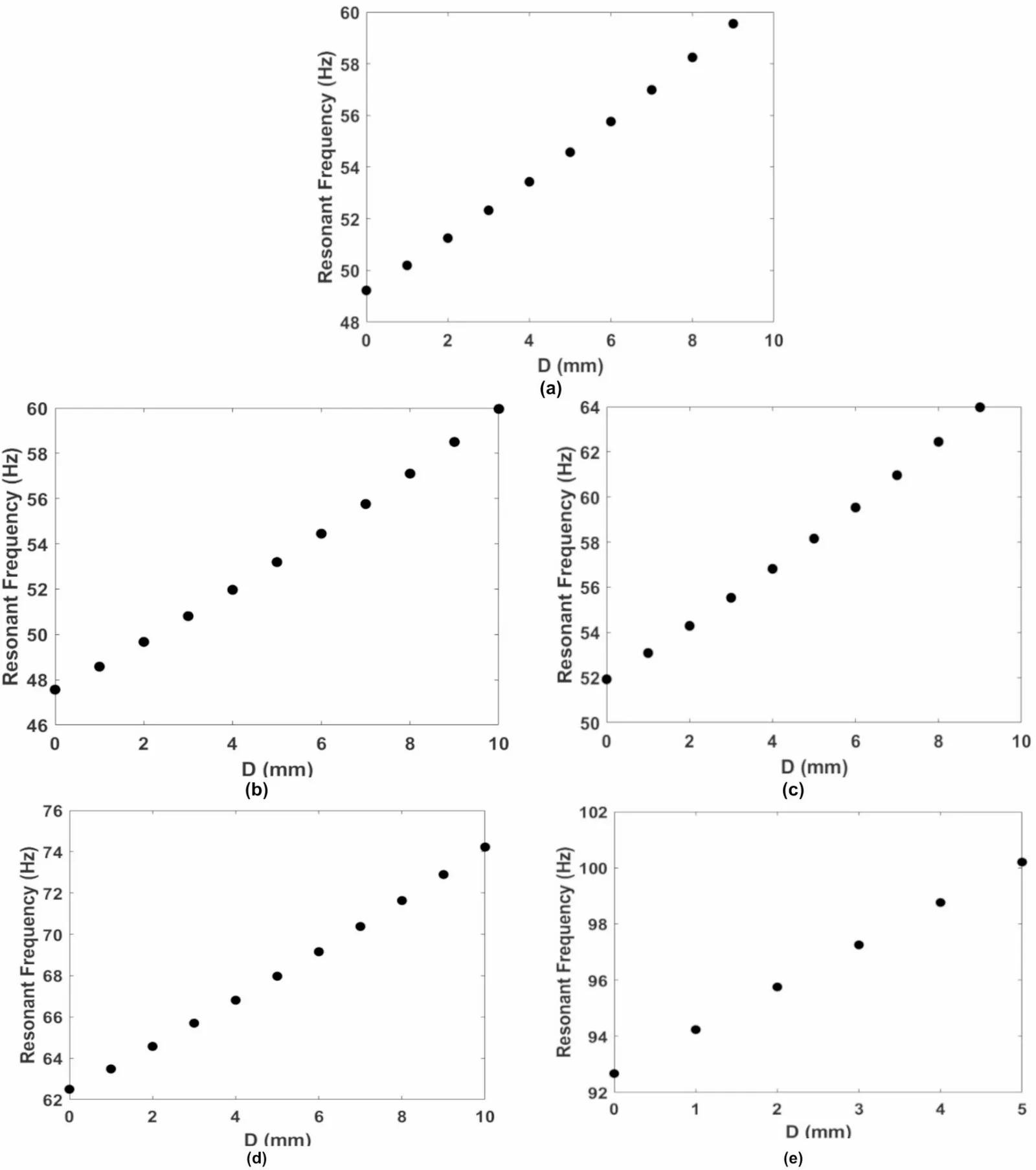
Simulation results of slot position (D) from fixed-end with resonant frequency, for the cantilever with (**a**) a rectangle slot, (**b**) a symmetric H-slot, (**c**) an asymmetric H-slot, (**d**) a triangle slot, and (**e**) an inverted triangle slot.

**Fig. 9:**
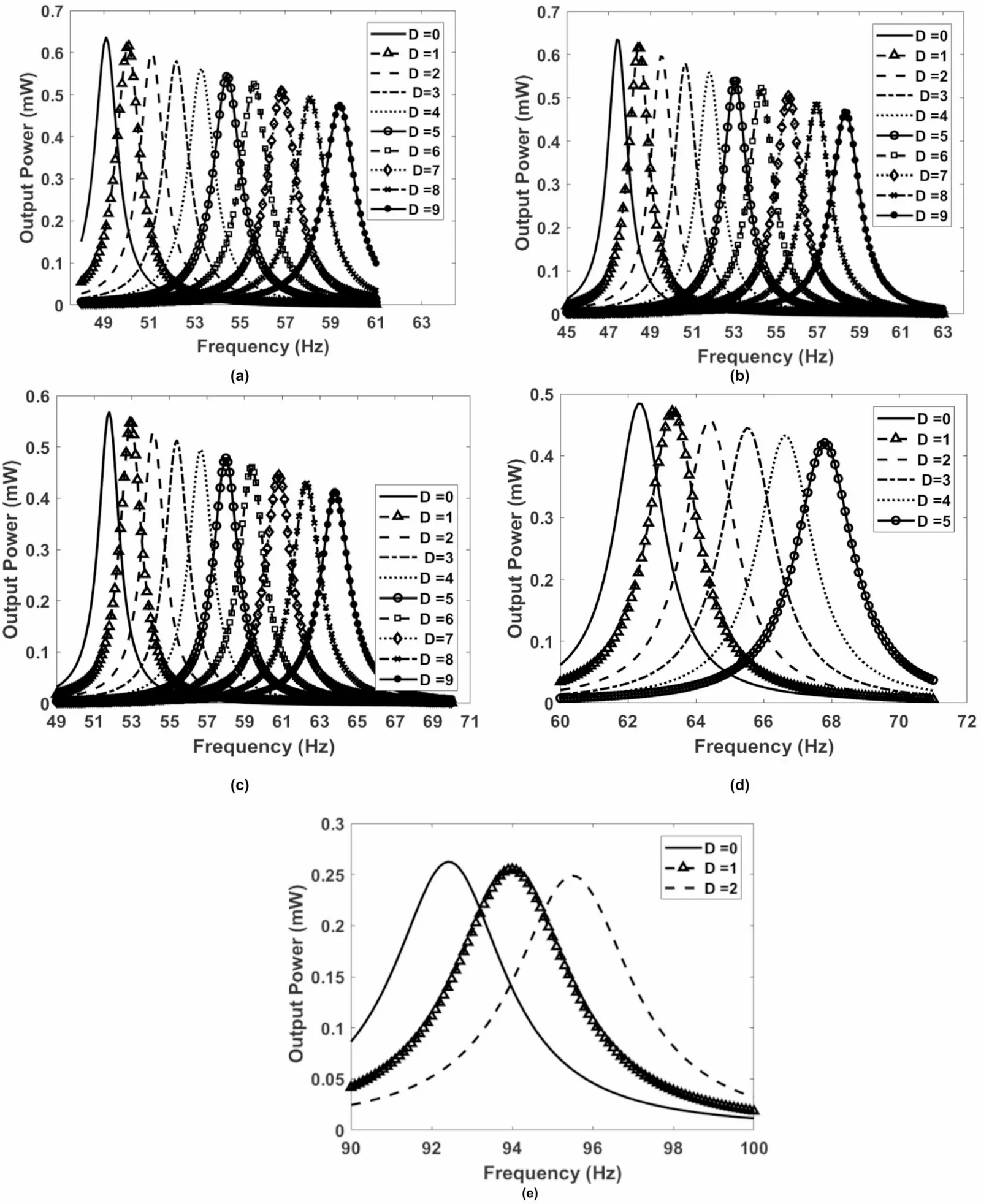
Simulation results of output power versus frequency for the cantilever with (**a**) a rectangle slot, (**b**) a symmetric H-slot, (**c**) an asymmetric H-slot, (**d**) a triangle slot, and (**e**) an inverted triangle slot.

**Fig. 10:**
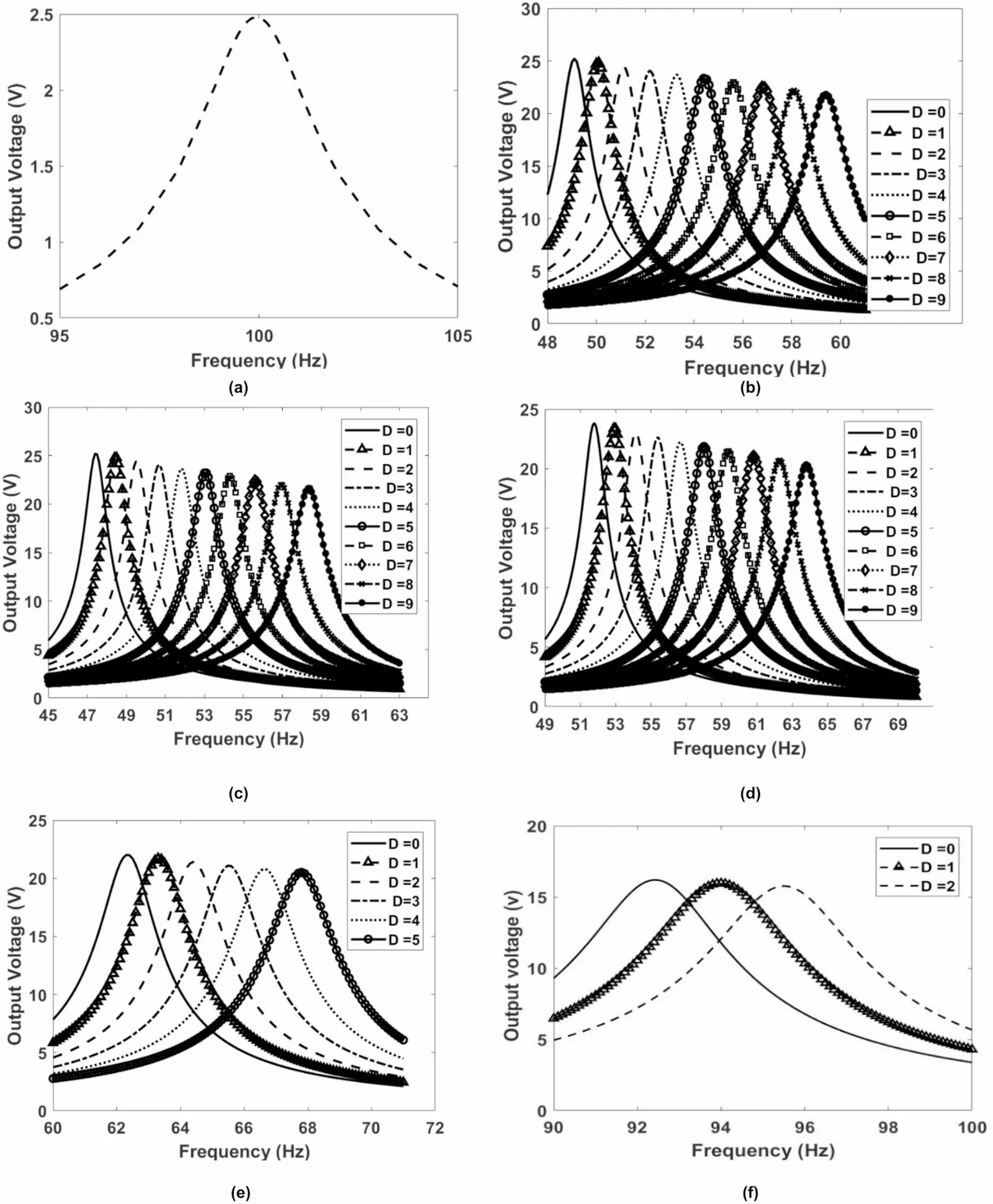
Numerical results of the output voltage versus frequency with different D for the cantilever with (**a**) no slot, (**b**) rectangular slot, (**c**) a symmetric H-slot, (**d**) an asymmetric H-slot, (**e**) a triangle slot, and (**f**) an inverted triangle slot.

**Fig. 11:**
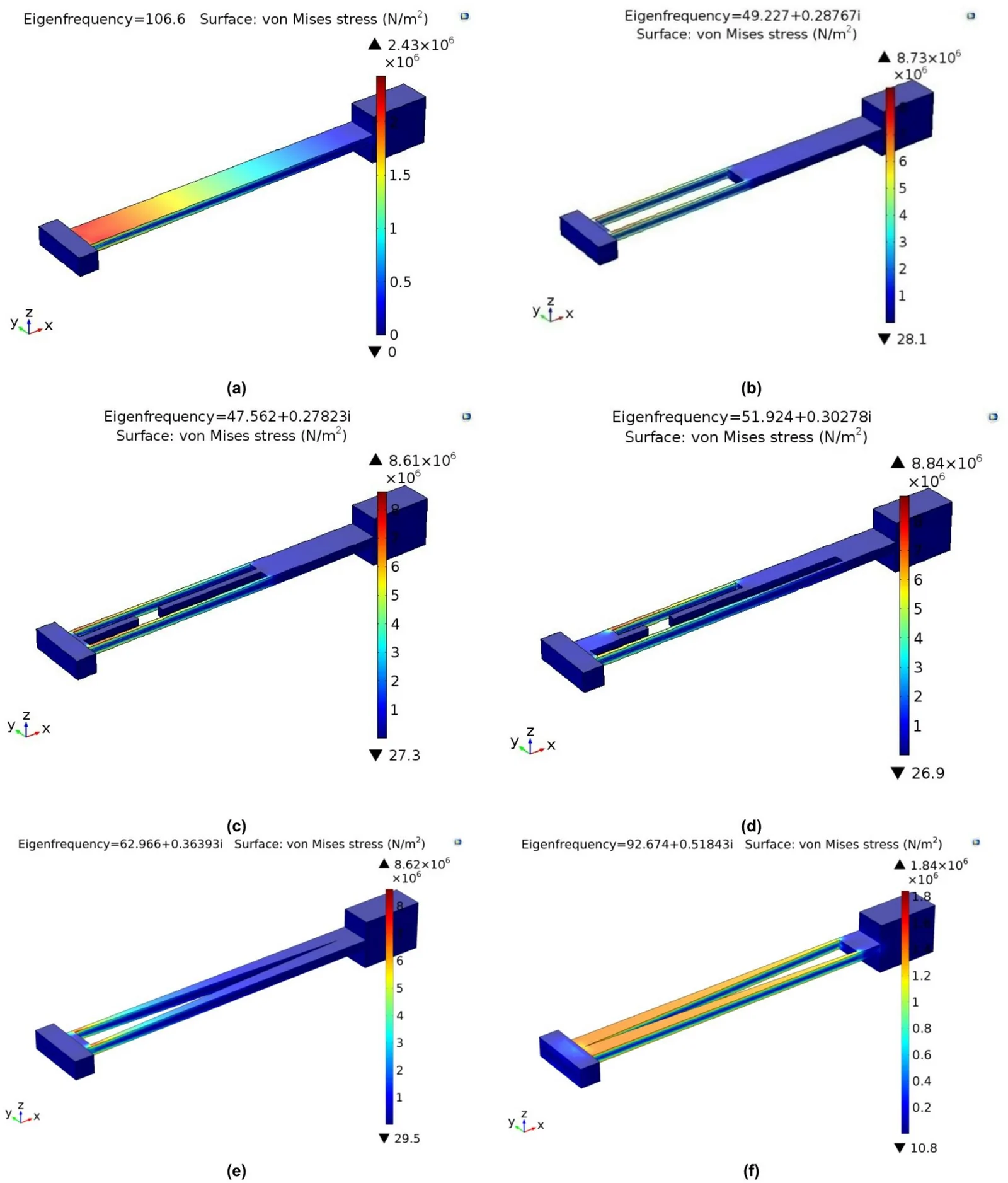
Distribution of von Mises stress along the cantilever at the resonance frequency for the cantilever with (**a**) no slot, (**b**) a rectangular slot, (**c**) a symmetric H-slot, (**d**) an asymmetric H-slot, (**e**) a triangle slot, and (**f**) an inverted triangle slot.

From the analysis in Fig. 7, the rectangular slot achieves its optimum performance at a length of 24 mm and a width of 4 mm. These dimensions are adopted for all other slot geometries to maintain fairness and consistency in the comparison. The influence of slot position on the resonant frequency is examined by varying the slot location (D) in steps of 1 mm from the fixed end for all proposed geometries, as illustrated in Fig. 8. It is observed that moving the slot away from the clamped end results in a higher resonant frequency. Among the studied configurations, the symmetric H-slot consistently exhibited the lowest resonant frequency at the same position compared with the other geometries. A comparative study is carried out to examine the effect of slot position on the electrical performance of the proposed cantilevers. The results, shown in Figs. 9 and 10 indicate that shifting the slot away from the fixed end leads to a gradual decrease in output voltage and power, while the resonant frequency increases. Nevertheless, the use of slots improves the performance compared with the solid cantilever. As illustrated in Fig. 10, the output voltage is increased by almost ten times, and the output power is nearly doubled. These observations are summarized in Table 2, where it is clear that the rectangular and symmetric H-slot geometries provide the most notable enhancement. The symmetric H-slot, in particular, achieved the lowest resonant frequency at the same slot position, making it a strong candidate for harvesting energy from low-frequency vibrations. The physical reason behind this improvement is explained by the stress distribution, as presented in Fig. 11. The solid beam recorded a maximum von Mises stress of 2.4 × 10⁶ N/m², while the rectangular and symmetric H-slots reached much higher levels of 8.7 × 10⁶ N/m² and 8.6 × 10⁶ N/m², respectively. Concentrating the stress near the clamped region increases the strain transferred to the piezoelectric layer, which directly accounts for the observed rise in voltage and power generation.

It should be emphasized that Eq. (13) is formulated within the framework of the Euler-Bernoulli beam theory, which assumes uniaxial bending and represents the slotted beam using an equivalent constant moment of inertia ($\:{I}_{eff}$). Consequently, the analytical model predicts a linear stress distribution over the beam cross-section. By contrast, the finite element results in Fig. 11 reveal a non-linear stress distribution because the Von Mises stress incorporates the combined effects of normal and shear stresses within a three-dimensional stress state. Moreover, the introduction of slots produces local stiffness discontinuities that alter the stress field, leading to pronounced stress concentrations and local shifts of the neutral axis in the vicinity of the slot boundaries. These inherently localized phenomena are rigorously captured by the finite element formulation but lie beyond the assumptions of the equivalent analytical model, whose objective is to describe the global structural behavior rather than resolve local stress gradients.

A comparative evaluation of the solid cantilever and various slotted beam designs is summarized in Table 2. The results indicate that the addition of slots markedly improves harvested power and power density compared to the solid reference beam. Among the examined configurations, the rectangular and symmetric H-slot designs provided the most substantial enhancement, nearly doubling the output of the baseline structure. These slot-based geometries also shift the resonant frequencies to lower values, making them more compatible with the low-frequency vibration conditions encountered in practical applications. In contrast, the triangular and inverted triangular slots provide only moderate improvement, whereas the asymmetric H-slot performs less effectively than its symmetric counterpart, highlighting the importance of a uniform stress distribution. These observations confirm that slot geometry is a decisive factor in balancing stiffness reduction and electromechanical efficiency. For clarity, the reported power densities of different piezoelectric energy harvesters are summarized in Table 3. In the field of piezoelectric energy harvesting (PEH), researchers report various performance metrics such as output voltage, output power, and power density. However, these values are highly dependent on the specific testing conditions, including excitation frequency, acceleration level, proof mass, and device volume. The performance of piezoelectric energy harvesters is often misrepresented by output power or power density alone, as these values depend on device geometry, excitation frequency, and applied acceleration. To enable fair comparison across different designs, the normalized power density (NPD) is used [68–70], defined by scaling the power density with a^2^f where a is the applied acceleration (g) and f the resonant frequency. This metric provides a consistent benchmark of intrinsic harvester efficiency, independent of testing conditions. As listed in Table 3, the (NPD) provides a fair basis for comparing harvesters tested under different excitation conditions. Among all reported configurations, the symmetric H-slot design achieved the highest NPD (0.54 mW·cm⁻³·g⁻²·Hz⁻¹), outperforming the closest literature benchmark (Xu et al. [43], NPD = 0.311 mW·cm⁻³·g⁻²·Hz⁻¹) by approximately 74%, despite operating at a higher resonant frequency (47 Hz vs. 23.9 Hz). The rectangular slot design followed closely (NPD = 0.52 mW·cm⁻³·g⁻²·Hz⁻¹), while the least effective geometry is the inverted triangular slot (NPD = 0.11 mW·cm⁻³·g⁻²·Hz⁻¹) and remains comparable to several literature designs.

**Table 2:** Comparison of output voltage, power, power density, and resonant frequency for the solid cantilever and various slotted geometries.

Type	Volume (mm³)	Output voltage (V)	Voltage decay rate (V/mm)	Output power (mW)	Power decay rate (mW/mm)	Power density (mW/cm³)	Resonant frequency (Hz)	Resonant frequency increasing rate (Hz/mm)
Solid beam	765	2.4	–	0.32	–	0.42	100	–
Rectangle slot	625.5	24.4	0.40	0.64	0.018	1.02	49	1.07
Symmetric H-slot	625.5	24.4	0.40	0.64	0.018	1.02	47	1.2
Asymmetric H-slot	625.5	24.3	0.32	0.56	0.015	0.89	51	1.3
Triangle slot	625.5	22.0	0.30	0.50	0.013	0.79	62	1.1
Inverted triangle slot	625.5	16.0	0.20	0.26	0.006	0.41	92	1.5

## Slotted cantilever arrays

**Fig. 12:**
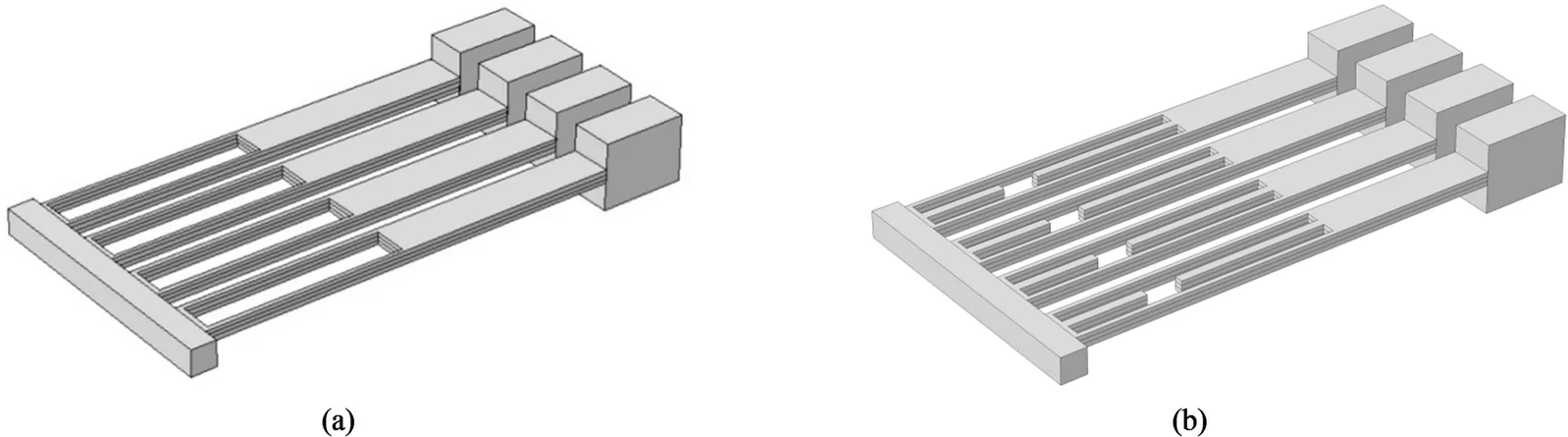
Geometrical configuration of the uniform slotted cantilever array: (**a**) rectangular slot and (**b**) symmetric H-slot.

This section presents the FEA model developed to investigate slotted cantilever arrays, followed by the corresponding results. A single cantilever generates useful power, but it is often insufficient to drive most practical applications. Arranging the harvesters in an array addresses this limitation, as the output increases with the number of units, making the system more suitable for powering electronic devices. Another advantage of the array configuration is its flexibility. By introducing small variations in the dimensions of each cantilever, the array responds over a wider frequency band and remains effective even when the excitation frequency changes. The simulation results show that the rectangular slot and symmetric H-slot designs achieve the best performance in array configurations. They exhibit lower resonant frequencies and higher stress concentrations, which lead to improved energy conversion. Overall, the slotted cantilever arrays provide a reliable approach for harvesting low-frequency vibrations and delivering stable energy in industrial environments (Fig. 12).

**Table 3:** A comparison of the power densities reported for different piezoelectric energy harvester.

References	Structures	Resonant freq. (Hz)	Accel. (g)	Power density (mW·cm⁻³)	NPD (mW·cm⁻³·g⁻²·Hz⁻¹)
Ibrahim et al.^41^	Thickness-tapered	156	1	0.10	0.664 × 10^− 3^
Xu et al.^43^	Trapezoidal hollow	23.9	1	7.24	0.311
Săvescu et al.^44^	Triangular beam	149.7	1	0.91	0. 610 × 10^− 2^
Wang et al.^47^	Trapezoidal slot	56.3	0.5	2.52	0.18
Mohiuddin et al.^48^	Inverted trapezoidal slot	42.4	1	2.73	0.064
Anand et al.^71^	Tapered multi-perforated beam	98.6	1	7.75	0.079
This work: solid	Cantilever (no slot)	100	0.2	0.42	0.10
This work: rectangular slot	Slotted cantilever	49	0.2	1.02	0.52
This work: sym. H-slot	Slotted cantilever	47	0.2	1.02	0.54
This work: asym. H-slot	Slotted cantilever	51	0.2	0.89	0.43
This work: triangle slot	Slotted cantilever	62	0.2	0.79	0.32
This work: inv. triangle slot	Slotted cantilever	92	0.2	0.41	0.11

### FEA for proposed cantilever array

**Fig. 13:**
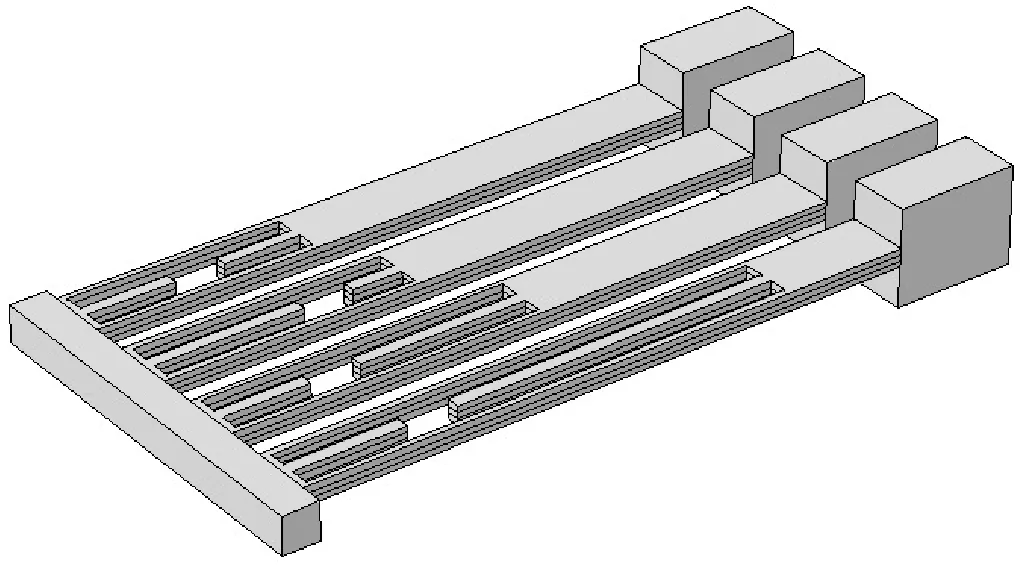
Geometrical configurations of the symmetric H-slot array with different slot lengths.

To evaluate the performance of the proposed array structures, a finite element model is developed. The analysis focused on the rectangular-slot and symmetric H-slot configurations, as these geometries exhibited the highest energy harvesting performance in the single-cantilever study. Each cantilever is modeled as a fixed-free structure subjected to harmonic base excitation, and its coupled electromechanical response is evaluated in terms of resonant frequency and harvested power. Two array configurations are considered. The first is a uniform array, in which all cantilevers have identical dimensions and therefore operate at the same resonant frequency. As shown in Fig. 12, rectangular slot and symmetric H-slot cantilevers are arranged side by side, with proof masses attached to their free ends to enhance the vibration response. The electrical outputs of all cantilevers are connected in parallel and supplied to a common resistive load. This arrangement is selected to increase the total harvested power by combining the current contributions from all array elements. A series connection, although capable of producing a higher output voltage, isn’t considered since voltage enhancement isn’t the primary objective of the present design. The second configuration is a broadband array, illustrated in Fig. 13, where the cantilevers are designed with different slot lengths to obtain a distributed set of resonant frequencies. This frequency distribution enables the array to harvest energy over a wider excitation bandwidth than the uniform configuration. The cantilevers are again connected in parallel because their electrical outputs vary across the operating frequency range. In addition, the parallel configuration is less affected by electrical mismatch among the array elements and allows each cantilever to contribute effectively when excited near its own resonant frequency while sharing the same load. The harvested power is calculated using$\:\:P={V}^{2}/{R}_{L}$, where V is the output voltage across the external load resistance $\:{R}_{L}$. In addition, the operating bandwidth is defined as the frequency range over which the harvested power remains greater than 50% of its maximum value. The incorporation of slots along the beam surface alters the stress distribution, creating localized regions of higher strain and improving the electromechanical interaction. The rectangular-slot version offers a straightforward geometry and is relatively easier to fabricate, while the H-slot arrangement introduces additional slotting that further increases stress concentration and lowers the resonant frequency. These distinct characteristics make both designs attractive for array applications.

### Array results

**Fig. 14:**
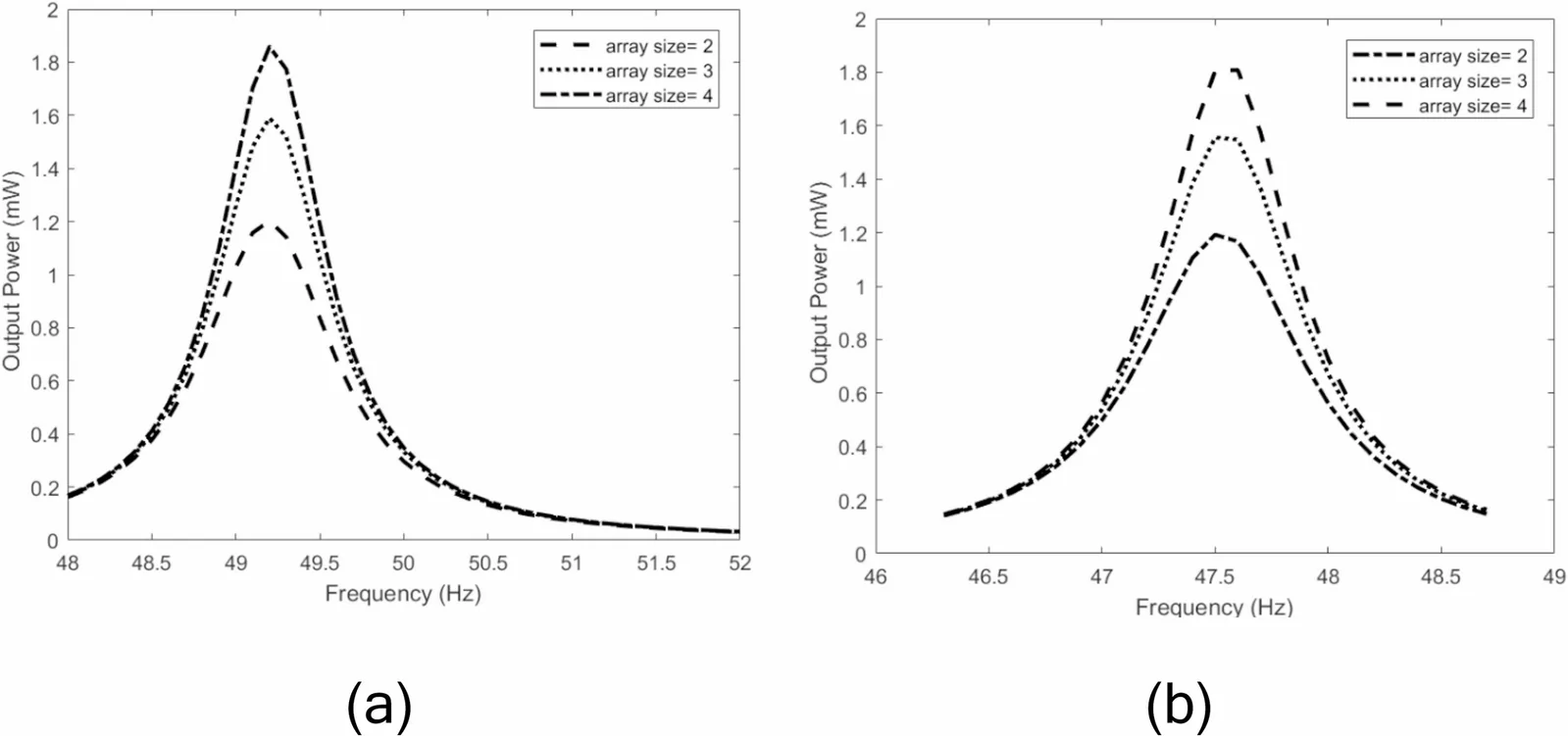
Output power versus frequency response for an array of the (**a**) rectangular slot and (**b**) symmetric H-slot, at different array sizes.

**Fig. 15:**
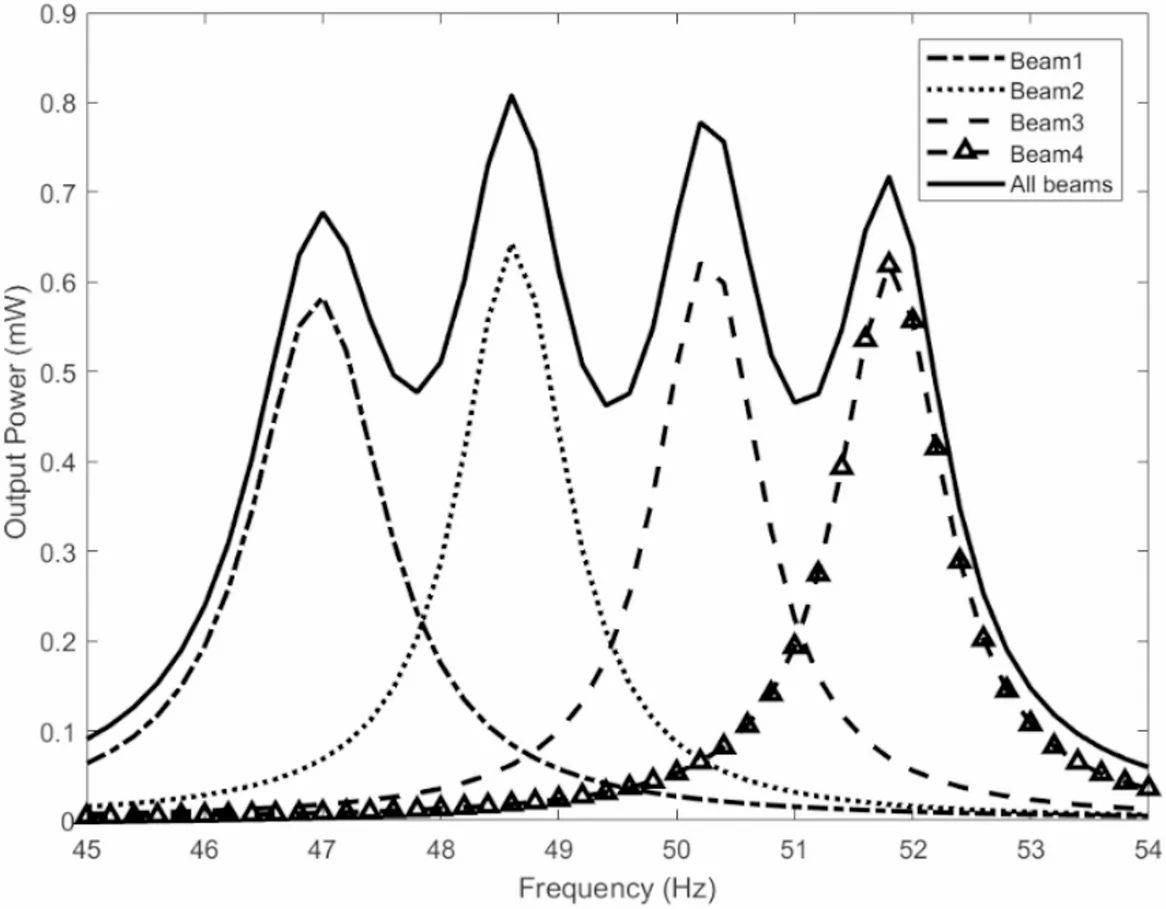
Output power versus frequency response for an array of symmetric H-slot cantilevers with different lengths.

The simulated performance of the proposed array configurations is summarized in Table 4. For the uniform arrays as shown in Fig. 12, increasing the number of cantilevers led to a corresponding increase in the harvested power. The rectangular-slot array achieved a maximum output power of 1.86 mW using four cantilevers, while the symmetric H-slot array reached 1.80 mW under the same conditions, as shown in Fig. 14. Despite the higher power output, both configurations exhibited relatively narrow operating bandwidths of 0.8 Hz and 1 Hz, respectively. The broadband array shown in Fig. 13 exhibited a different performance. In this configuration, the cantilevers are designed with different slot lengths, resulting in different resonant frequencies and a broader operating frequency range. Although the maximum harvested power decreases to 0.8 mW, the bandwidth increases significantly to 5.8 Hz. In addition, the harvested power remained above 50% of its maximum value throughout this frequency range. These results demonstrate the different roles of the two array configurations. The uniform arrays are intended to maximize the harvested power by combining the outputs of identical cantilevers operating at the same resonant frequency. In contrast, the broadband array sacrifices part of the peak power to achieve a much wider operating bandwidth, allowing effective energy harvesting over a broader range of excitation frequencies. This makes the broadband configuration more suitable for applications where the vibration frequency varies with time, whereas the uniform arrays are better suited to environments with nearly constant excitation frequencies (Fig. 15).

**Table 4:** Comparison of output power and bandwidth for different slotted cantilever array configurations.

Type	Array size	Output power (mW)	Bandwidth (Hz)
An array of cantilevers with a symmetric H–slot with same dimensions	2	1.19	0.9
	3	1.55	1
	4	1.80	1
An array of cantilevers with the same dimension Rectangle slot	2	1.2	0.8
	3	1.59	0.8
	4	1.86	0.8
An array of cantilevers with different lengths, symmetric H–slot	4	0.8	5.8

## Conclusion

This study examined the effect of slot shape on the performance of the piezoelectric cantilever energy harvesters through analytical modeling and finite element analysis. Different slot geometries incorporating a rectangle slot, a symmetric H-slot, an asymmetric H-slot, a triangle slot, and an inverted triangle slot were developed. All configurations were designed with identical external dimensions, proof masses, and material properties. In addition, the same material volume 139.5 mm³ is removed from each cantilever, ensuring that the observed performance variations were solely associated with the slot shape. The results showed that both slot dimensions and slot location play a significant role in determining the electromechanical response of the harvester. Increasing the slot width produced a more pronounced improvement in performance than increasing the slot length, while locating the slot closer to the fixed end yielded the most favorable results. Among the geometries investigated, the symmetric H-slot configuration exhibited the best overall performance. Relative to the conventional solid cantilever, this design increased the harvested power from 0.32 mW to 0.64 mW, raised the output voltage from 2.4 V to 24.4 V, and reduced the resonant frequency from 100 Hz to 47 Hz. The lower resonant frequency improves compatibility with low-frequency ambient vibration sources, which are commonly encountered in practical environments. To further increase the harvested energy, array structures based on the rectangular and symmetric H-slot were implemented. The harvested power increased with the number of elements, reaching 1.86 mW with bandwidth of 0.8 Hz for the rectangular slot array and 1.80 mW with bandwidth 1 Hz for the symmetric H-slot array. These configurations provided the highest power output but remained limited by a relatively narrow operating bandwidth. While arrays of varied-length H-slots expanded the bandwidth to 5.8 Hz, keeping output above 50% of the peak. Thus, uniform arrays maximize power, whereas varied arrays improve robustness to frequency shifts. The proposed designs have been validated using the 3D FEM numerical simulation and showed excellent performance, surpassing literature configurations.

As a continuation of this work, the optimized harvester will be fabricated and experimentally tested to validate the numerical predictions under practical operating conditions.

## Data Availability

All data in support of the findings of this paper are available within the article.
